# Interferon-gamma to interleukin-10 ratio is negatively associated with short-term mortality risk in sepsis patients: a retrospective study based on immune balance index

**DOI:** 10.3389/fimmu.2026.1816500

**Published:** 2026-07-28

**Authors:** Mingyuan Pan, Wenhui Huang, Yuyang Li, Hongsheng Lu, Xiao Teng, Zheng Li

**Affiliations:** 1 The Second Affiliated Hospital of Guangxi Medical University, Nanning, Guangxi, China; 2 Wuming Hospital of Guangxi Medical University, Nanning, Guangxi, China; 3 The First Affiliated Hospital of Guangxi Medical University, Nanning, Guangxi, China

**Keywords:** immunity, interferon-γ, interleukin-10, prognosis, sepsis

## Abstract

**Background:**

Sepsis is a life-threatening organ dysfunction caused by a dysregulated host response to infection. Its immunopathology is characterized by a dynamic imbalance between pro-inflammatory and anti-inflammatory responses. Interferon-γ (IFN-γ) and interleukin-10 (IL-10) are representative pro- and anti-inflammatory mediators, respectively, and their ratio (IIR) may provide a more sensitive reflection of immune balance in patients with sepsis. However, most previous studies have focused on the prognostic value of individual cytokines, and the association between IIR and short-term mortality risk, as well as its predictive performance, remains unclear.

**Methods:**

We retrospectively analyzed clinical data from 411 patients with sepsis admitted to the intensive care unit (ICU) of the Second Affiliated Hospital of Guangxi Medical University between December 2023 and January 2026. Patients were divided into low, intermediate, and high IIR groups according to tertiles of the first IIR value measured within 24 h after ICU admission. Kaplan-Meier curves were generated to compare survival across groups using the log-rank test. Multivariable Cox proportional hazards regression models were used to evaluate the association between IIR and mortality risk. Restricted cubic spline analysis was performed to explore potential dose-response relationships, followed by threshold effect analysis. Receiver operating characteristic curves were constructed to assess the predictive value of IIR, and subgroup analyses were conducted to examine the robustness of the findings.

**Results:**

A total of 411 patients were included. Kaplan-Meier analysis showed that patients with higher IIR levels had significantly lower risks of 14-day and 28-day all-cause mortality (log-rank P < 0.001). After adjustment for confounders, multivariable Cox regression confirmed an independent negative association between high IIR levels and both 14-day mortality (HR = 0.48, 95% CI: 0.30–0.77, P < 0.05) and 28-day mortality (HR = 0.62, 95% CI: 0.42–0.90, P < 0.05). Restricted cubic spline analysis revealed a nonlinear association between IIR and short-term mortality. Two-piecewise Cox regression identified inflection points at 0.066 for 14-day mortality and 0.073 for 28-day mortality. Receiver operating characteristic curve analysis indicated that IIR had favorable predictive value for short-term mortality in patients with sepsis. Subgroup analyses yielded consistent associations, with no significant interaction observed across predefined subgroups.

**Conclusion:**

The IFN-γ/IL-10 ratio was nonlinearly and negatively associated with 14-day and 28-day mortality risk in patients with sepsis. Higher IIR levels were significantly associated with reduced short-term mortality, suggesting that this immune balance index may serve as an independent predictor of short-term prognosis.

## 1 Introduction

Sepsis is a life-threatening organ dysfunction caused by a dysregulated host response to infection and remains one of the leading causes of death among patients admitted to intensive care units (1). Its pathophysiology involves complex immune dysregulation, typically characterized by an early hyperinflammatory response followed by immunosuppression (2–4), which increases susceptibility to secondary infections and contributes to poor outcomes. Biomarkers that accurately reflect immune status and help predict prognosis are therefore of considerable clinical importance (5).

Current prognostic assessment in sepsis relies mainly on single inflammatory mediators, such as interleukin-6, interleukin-10, and tumor necrosis factor-α, or their combination with clinical scoring systems, including the Acute Physiology and Chronic Health Evaluation score (6–11). Yet the immune response in sepsis is dynamic and involves continuous shifts between pro-inflammatory and anti-inflammatory mechanisms. A single cytokine is unlikely to capture the overall balance of host immunity, whereas multi-marker models, despite improving predictive performance, may increase the complexity of clinical application.

In this context, the ratio between pro-inflammatory and anti-inflammatory cytokines may offer a more integrated representation of immune status in sepsis.

IFN-γ is an important pro-inflammatory cytokine involved in host defense and pathogen clearance (12). A reduced IFN-γ response in patients with sepsis has been associated with poor outcomes and an immunosuppressive phenotype (13, 14). IL-10, a key anti-inflammatory cytokine, is generally released later than pro-inflammatory mediators and participates in negative feedback regulation by suppressing pro-inflammatory cytokine production and downregulating human leukocyte antigen-DR expression on monocytes, which is considered a marker of immunosuppression (15). The IFN-γ/IL-10 ratio reflects the dynamic balance between T helper 1-type immune responses and regulatory T-cell/anti-inflammatory responses, and may therefore provide a more comprehensive assessment of host immune function (16). By integrating both pro- and anti-inflammatory signals, this ratio may overcome the limitations of single-cytokine thresholds and allow a more precise evaluation of immune homeostasis.

At present, evidence regarding the prognostic value of IIR in sepsis remains exploratory. This study aimed to investigate the predictive value of IIR for short-term all-cause mortality in patients with sepsis and to further characterize its nonlinear association with mortality risk, threshold effect, and consistency across different subgroups, with the goal of providing a potential biomarker for precision immunomodulation in sepsis.

## 2 Methods

### 2.1 Data source

This retrospective study was based on data from patients with sepsis admitted to the Department of Critical Care Medicine, Second Affiliated Hospital of Guangxi Medical University, between December 2023 and January 2026. All biochemical data were obtained from routine clinical laboratory records in the hospital electronic medical record system and laboratory information management system. The measurements were derived from venous blood samples collected within 24 h after ICU admission as part of standard clinical care.

### 2.2 Study population

**Figure 1:**
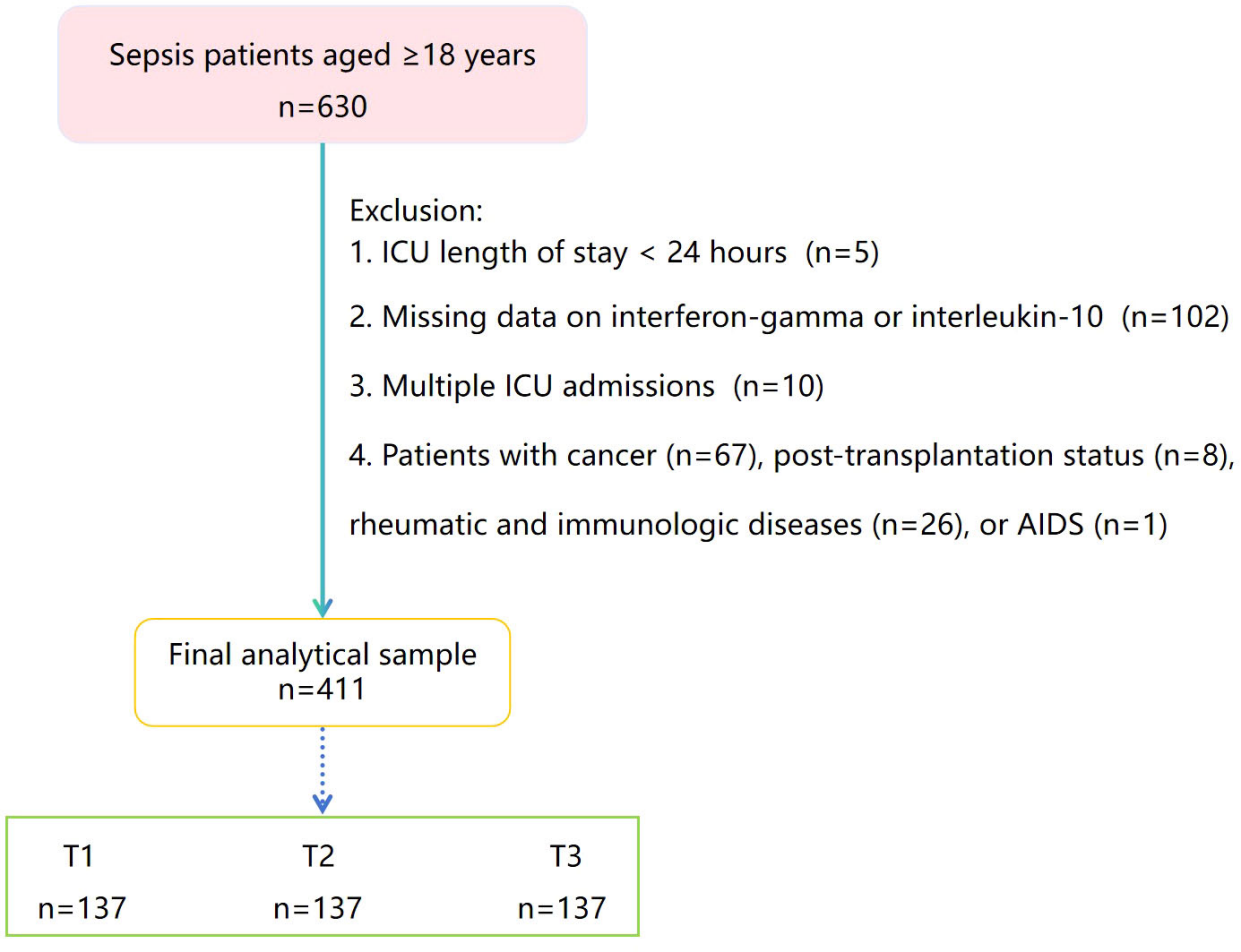
Patient enrollment flow.

Hospitalization records of patients with sepsis admitted between December 2023 and January 2026 were reviewed. To ensure the robustness of the analysis, strict eligibility criteria were applied. Patients were included if they met the Sepsis 3.0 diagnostic criteria (1), were aged ≥18 years, had an ICU stay longer than 24 h, and had complete IFN-γ and IL-10 data. For patients with multiple ICU admissions, only the first admission was included. Patients were excluded if they were aged <18 years, had an ICU stay <24 h, lacked IFN-γ or IL-10 data, or had malignant tumors, a history of organ transplantation, rheumatic or autoimmune diseases, or acquired immunodeficiency syndrome. Ultimately, 411 patients were included in the study (**Figure 1**).

### 2.3 Handling of outliers and missing values

Outliers were processed using the winsor2 command in STATA with 1% and 99% truncation points. Missing data were handled by multiple imputation. Variables with more than 15% missing values were excluded, and the remaining variables were imputed using multiple imputation.

### 2.4 Grouping and clinical outcomes

IIR was calculated as follows: IIR = IFN-γ (pg/mL)/IL-10 (pg/mL) (17). Both cytokines were measured from the same specimen and within the same assay batch. Because the numerator and denominator had the same unit (pg/mL), the units were canceled during ratio calculation; thus, IIR is a dimensionless ratio rather than an index with an independent physical unit. Detailed assay information is provided in the **Supplementary Material**. Patients were divided into three groups according to IIR tertiles. The study endpoints were 14-day and 28-day all-cause mortality after ICU admission.

### 2.5 Ethical statement

This retrospective case analysis was conducted in accordance with the Declaration of Helsinki and relevant Chinese regulations on ethical review of biomedical research involving human participants. The study was approved by the Ethics Review Committee of the Second Affiliated Hospital of Guangxi Medical University. Because this study involved only secondary analysis of previously collected clinical data, did not include any intervention, and all patient information was fully de-identified before analysis, the requirement for informed consent was waived by the ethics committee.

### 2.6 Statistical analysis

Continuous variables were presented as median and interquartile range (IQR) for non-normally distributed data. Between-group comparisons were performed using the Mann-Whitney U test or Kruskal-Wallis test, as appropriate. Categorical variables were summarized as frequencies and percentages and compared using the chi-square test or Fisher’s exact test.Patients were stratified into three groups according to IIR tertiles. Kaplan-Meier curves were used to describe survival trajectories for the primary endpoints, and differences among groups were assessed using the log-rank test. Univariate analysis, LASSO regression, and multivariable Cox proportional hazards regression models were applied to evaluate the association between IIR, treated as a categorical variable, and 14-day and 28-day all-cause mortality. Multivariable models were adjusted for potential confounders, including age, sex, SOFA score, APACHE II score, interleukin-6, total bilirubin, lactate, anion gap, international normalized ratio, creatine kinase-MB, mechanical ventilation, and antihypertensive therapy.Restricted cubic spline analysis based on Cox regression was used to explore potential nonlinear relationships between IIR and mortality. A two-piecewise Cox proportional hazards model was further applied to assess threshold effects and identify inflection points. Receiver operating characteristic curves were generated to evaluate the predictive ability of IIR for all-cause mortality at different time points, and the area under the curve was calculated. Decision curve analysis and calibration curves were also performed. Subgroup analyses were conducted to assess the consistency of the association between IIR and mortality across predefined subgroups, including sex, age, hypertension, acute kidney injury, pneumonia, diabetes, heart failure, continuous renal replacement therapy, mechanical ventilation, vasoactive agents, corticosteroids, antihypertensive drugs, immunosuppressants, and Candida infection. Further details are provided in the **Supplementary Material**.

All analyses were performed using the DecisionLinnc analysis platform (DecisionLinnc Core Team, 2023, Hangzhou, China; available at https://www.statsape.com/). All statistical tests were two-sided, and P < 0.05 was considered statistically significant.

## 3 Results

### 3.1 Patient characteristics

A total of 411 patients diagnosed with sepsis were included. The median age was 62.00 years (IQR, 52.00–74.00), and 198 patients (48.18%) were female. ICU mortality, 14-day all-cause mortality, and 28-day all-cause mortality were 20.44%, 34.79%, and 47.20%, respectively.According to IIR tertiles, patients were classified into low (T1), intermediate (T2), and high (T3) IIR groups. Baseline characteristics differed significantly among the three groups. Pulmonary infection was the predominant source of infection in both the high- and low-IIR groups. Patients in the high-IIR group showed higher proportions of Acinetobacter baumannii, Klebsiella pneumoniae, Staphylococcus aureus, and fungal infections, whereas Escherichia coli and other pathogens were relatively more frequent in the low-IIR group, although these distributional differences did not reach statistical significance (all P>0.05). Hypertension, diabetes, and pneumonia were more prevalent in the high-IIR group, while acute kidney injury, chronic kidney disease, heart failure, and coronary heart disease were more common in the low-IIR group. In addition, patients in the high-IIR group had lower lactate levels, cytokine levels, SOFA scores, and APACHE II scores, and were less frequently treated with continuous renal replacement therapy, mechanical ventilation, and corticosteroids. Compared with the low-IIR group, the high-IIR group had lower mortality at all time points. The 14-day all-cause mortality rates were 49.46%, 35.04%, and 19.71% across the low, intermediate, and high groups, respectively (P < 0.001). The corresponding 28-day all-cause mortality rates were 63.50%, 43.07%, and 35.04% (P < 0.001) (**Table 1**).

**Table 1:** Baseline characteristics.

Variable	Overall N = 411	T1 = 137	T2 = 137	T3 = 137	P value
IIR	0.24 (0.04 - 0.66)	0.02 (0.01 - 0.04)	0.24 (0.14 - 0.36)	0.97 (0.66 - 1.24)	<0.001
NLR	12.77 (7.03 - 23.42)	12.96 (8.07 - 23.16)	12.53 (6.83 - 21.28)	12.61 (6.10 - 24.92)	0.521
PLR	232.73 (120.14 - 433.33)	194.20 (84.15 - 423.38)	215.07 (140.00 - 414.63)	294.00 (141.53 - 458.54)	0.004
SII	2,209.32 (946.18 - 4,680.86)	2,114.87 (658.00 - 4,592.63)	2,085.00 (964.63 - 4,219.84)	2,483.14 (1,140.57 - 5,127.82)	0.148
Demographics					
Age, year	62.00 (52.00 - 74.00)	62.00 (52.00 - 73.00)	62.00 (52.00 - 74.00)	63.00 (52.00 - 74.00)	0.743
Gender, n(p%)					0.481
Male	198.00 (48.18%)	66.00 (48.18%)	71.00 (51.82%)	61.00 (44.53%)	
Female	213.00 (51.82%)	71.00 (51.82%)	66.00 (48.18%)	76.00 (55.47%)	
Comorbidities					
Hypertension, n(p%)	213.00 (51.82%)	70.00 (51.09%)	67.00 (48.91%)	76.00 (55.47%)	0.541
Acute Kidney Injury, n (%)	121.00 (29.44%)	55.00 (40.15%)	37.00 (27.01%)	29.00 (21.17%)	0.002
Chronic Kidney Disease, n (%)	55.00 (13.38%)	25.00 (18.25%)	15.00 (10.95%)	15.00 (10.95%)	0.123
Diabetes, n (%)	148.00 (36.01%)	50.00 (36.50%)	48.00 (35.04%)	50.00 (36.50%)	0.959
Ischemic Heart Disease, n (%)	56.00 (13.63%)	21.00 (15.33%)	16.00 (11.68%)	19.00 (13.87%)	0.675
Heart Failure, n (%)	83.00 (20.19%)	34.00 (24.82%)	25.00 (18.25%)	24.00 (17.52%)	0.253
Pneumonia, n (%)	398.00 (96.84%)	132.00 (96.35%)	131.00 (95.62%)	135.00 (98.54%)	0.457
Scores					
SOFA Score	9.00 (7.00 - 12.00)	11.00 (8.00 - 14.00)	9.00 (7.00 - 12.00)	8.00 (6.00 - 10.00)	<0.001
APACHE II Score	31.00 (25.00 - 37.00)	34.00 (27.00 - 39.00)	30.00 (24.00 - 36.00)	30.00 (23.00 - 35.00)	<0.001
Vital Signs					
Heart Rate, bpm	102.00 (88.00 - 120.00)	106.00 (90.00 - 123.00)	102.00 (88.00 - 120.00)	100.00 (88.00 - 113.00)	0.118
Respiratory Rate, bpm	20.00 (16.00 - 25.00)	20.00 (16.00 - 25.00)	21.00 (16.00 - 24.00)	20.00 (16.00 - 24.00)	0.961
Systolic Blood Pressure, mmHg	122.00 (103.00 - 145.00)	112.00 (96.00 - 140.00)	124.00 (107.00 - 142.00)	129.00 (108.00 - 155.00)	<0.001
Diastolic Blood Pressure, mmHg	65.00 (55.00 - 75.00)	61.00 (51.00 - 70.00)	65.00 (55.00 - 75.00)	68.00 (59.00 - 80.00)	0.001
Mean Arterial Pressure, mmHg	82.00 (70.00 - 94.00)	74.50 (66.00 - 93.00)	82.00 (71.00 - 91.00)	84.50 (76.00 - 100.00)	<0.001
Temperature, °C	37.00 (36.60 - 37.80)	37.00 (36.70 - 37.80)	37.00 (36.60 - 37.80)	37.00 (36.60 - 37.90)	0.714
Laboratory parameters					
White blood cells, 109/L	12.00 (8.51 - 17.25)	13.06 (9.11 - 19.00)	11.40 (7.83 - 16.19)	11.87 (8.99 - 16.55)	0.175
Red blood cells, 109/L	3.30 (2.53 - 4.05)	3.18 (2.37 - 3.91)	3.27 (2.56 - 3.95)	3.46 (2.62 - 4.14)	0.292
Hemoglobin, g/L	93.00 (71.00 - 117.00)	89.00 (68.00 - 115.00)	90.00 (71.00 - 116.00)	99.00 (73.00 - 118.00)	0.534
Platelets, 109/L	184.00 (109.00 - 284.00)	154.00 (77.00 - 262.00)	180.00 (129.00 - 285.00)	213.00 (147.00 - 288.00)	<0.001
Neutrophil cells,109/L	9.93 (6.74 - 15.02)	11.06 (6.91 - 17.14)	9.63 (6.06 - 12.84)	9.88 (7.64 - 14.05)	0.165
Lymphocyte cells, 109/L	0.76 (0.47 - 1.22)	0.79 (0.47 - 1.22)	0.72 (0.47 - 1.27)	0.76 (0.47 - 1.18)	0.933
Total bilirubin,μmol/L	14.10 (8.70 - 29.60)	17.80 (10.80 - 48.10)	12.10 (8.00 - 23.65)	12.35 (7.75 - 20.10)	<0.001
Direct bilirubin,μmol/L	7.30 (4.25 - 18.65)	10.50 (5.30 - 34.90)	6.45 (4.00 - 15.90)	6.60 (3.60 - 10.70)	<0.001
Indirect bilirubin,μmol/L	5.70 (3.50 - 11.30)	7.00 (3.90 - 14.70)	5.50 (3.30 - 11.00)	5.25 (3.30 - 8.60)	0.008
Albumin,g/L	29.10 (25.90 - 33.70)	28.90 (25.10 - 34.50)	28.30 (25.40 - 32.80)	30.20 (27.40 - 34.10)	0.039
Alanine aminotransferase,U/L	26.00 (15.00 - 59.00)	35.00 (16.00 - 89.00)	25.00 (16.00 - 52.00)	22.00 (14.00 - 41.00)	0.013
Aspartate aminotransferase,U/L	43.00 (25.00 - 97.00)	55.00 (29.00 - 187.00)	40.00 (25.00 - 87.00)	33.00 (24.00 - 62.00)	<0.001
Creatinine, mg/dL	148.00 (78.00 - 311.50)	190.00 (97.00 - 380.00)	149.00 (78.00 - 302.00)	113.00 (71.00 - 274.00)	0.002
Blood urea, mg/dL	12.62 (6.67 - 21.01)	13.81 (7.27 - 25.67)	13.93 (7.37 - 20.52)	10.15 (6.19 - 17.63)	0.039
Anion gap, mmol/L	9.90 (7.00 - 14.40)	13.30 (7.60 - 18.90)	9.50 (7.00 - 13.00)	8.65 (6.70 - 12.00)	<0.001
Calcium, mmol/L	1.04 (0.97 - 1.11)	1.03 (0.95 - 1.10)	1.03 (0.96 - 1.10)	1.05 (0.99 - 1.11)	0.036
Potassium, mmol/L	3.70 (3.30 - 4.20)	3.80 (3.40 - 4.50)	3.70 (3.20 - 4.20)	3.60 (3.30 - 4.00)	0.035
Sodium, mmol/L	140.00 (135.00 - 146.00)	141.00 (135.00 - 146.00)	140.00 (135.00 - 146.00)	140.00 (136.00 - 145.00)	0.988
Glucose, mmol/L	8.00 (6.10 - 11.60)	8.25 (5.85 - 13.30)	8.00 (6.30 - 11.80)	8.00 (6.20 - 10.40)	0.744
Neutrophil gelatinase associated lipocalin,ng/mL	275.00 (116.00 - 662.00)	426.00 (171.00 - 1,004.00)	289.00 (117.00 - 513.00)	208.00 (94.00 - 462.00)	<0.001
Heparin binding protein,ng/mL	42.80 (17.40 - 105.30)	57.50 (28.90 - 151.30)	40.50 (18.80 - 103.50)	31.80 (15.50 - 78.80)	<0.001
Lactate,mmol/L	1.70 (1.10 - 3.00)	2.60 (1.40 - 7.10)	1.50 (1.00 - 2.40)	1.40 (0.90 - 2.20)	<0.001
Creatine Kinase,ng/mL	143.00 (51.00 - 564.00)	217.00 (77.00 - 1,097.00)	144.00 (48.00 - 450.00)	104.00 (42.00 - 341.00)	<0.001
Creatine Kinase MB,ng/mL	19.00 (11.00 - 37.00)	24.00 (13.00 - 52.00)	18.00 (10.00 - 30.00)	16.00 (10.00 - 26.00)	<0.001
hs_cTnT, ng/ml	0.07 (0.03 - 0.19)	0.11 (0.05 - 0.24)	0.07 (0.03 - 0.18)	0.05 (0.02 - 0.14)	<0.001
proBNP, pg/ml	2,901.00 (774.00 - 12,348.00)	6,408.00 (1,678.00 - 17,747.00)	2,480.00 (636.00 - 13,245.00)	1,778.00 (370.00 - 6,865.00)	<0.001
PCT, ng/ml	2.36 (0.56 - 13.00)	5.91 (1.14 - 27.90)	2.44 (0.58 - 16.40)	1.21 (0.29 - 4.27)	<0.001
IL6, pg/ml	139.15 (38.70 - 511.00)	234.00 (111.00 - 1,499.00)	119.11 (40.70 - 426.34)	64.30 (26.60 - 312.00)	<0.001
IL4, pg/ml	1.61 (1.50 - 2.74)	1.93 (1.50 - 4.50)	1.68 (1.50 - 2.46)	1.50 (1.50 - 1.98)	<0.001
IL8, pg/ml	12.04 (4.57 - 62.29)	33.41 (8.18 - 219.04)	15.30 (5.60 - 57.42)	6.81 (3.46 - 15.28)	<0.001
IL10, pg/ml	10.75 (2.99 - 72.40)	142.31 (55.12 - 416.22)	9.60 (4.50 - 17.87)	2.31 (1.50 - 3.23)	<0.001
IL17, pg/ml	3.94 (2.45 - 10.90)	6.34 (2.45 - 18.62)	4.76 (2.59 - 11.37)	3.24 (2.25 - 5.04)	<0.001
IFN-a, pg/ml	1.88 (1.50 - 3.20)	1.87 (1.50 - 5.18)	1.87 (1.50 - 3.49)	1.90 (1.50 - 2.39)	0.866
IFN-γ, pg/ml	1.59 (1.50 - 3.68)	1.71 (1.50 - 3.70)	1.57 (1.50 - 3.39)	1.54 (1.50 - 4.50)	0.683
International normalized ratio,%	1.24 (1.11 - 1.47)	1.31 (1.13 - 1.59)	1.27 (1.13 - 1.49)	1.17 (1.07 - 1.31)	<0.001
Prothrombin time, s	13.70 (12.20 - 16.20)	14.40 (12.40 - 17.60)	14.00 (12.40 - 16.40)	12.80 (11.80 - 14.50)	<0.001
Partial thromboplastin time, s	33.30 (29.70 - 38.80)	34.50 (30.60 - 43.00)	34.00 (30.10 - 39.10)	31.80 (28.20 - 36.30)	<0.001
PH	7.40 (7.32 - 7.45)	7.36 (7.26 - 7.44)	7.40 (7.34 - 7.45)	7.41 (7.35 - 7.46)	<0.001
PaO2	114.00 (85.00 - 155.00)	112.00 (84.30 - 160.00)	120.00 (84.20 - 149.00)	108.00 (87.10 - 156.00)	0.946
PCO2	34.50 (29.90 - 40.10)	33.60 (27.90 - 39.50)	34.50 (29.80 - 40.20)	36.30 (30.90 - 40.20)	0.077
Treatments					
Continuous Renal Replacement Therapy, n (%)	140.00 (34.06%)	65.00 (47.45%)	41.00 (29.93%)	34.00 (24.82%)	<0.001
Ventilation, n (%)	354.00 (86.13%)	124.00 (90.51%)	118.00 (86.13%)	112.00 (81.75%)	0.111
Glucocorticoids, n (%)	164.00 (39.90%)	67.00 (48.91%)	52.00 (37.96%)	45.00 (32.85%)	0.021
Vasopressors, n (%)	331.00 (80.54%)	124.00 (90.51%)	105.00 (76.64%)	102.00 (74.45%)	0.001
Antihypertensive, n (p%)	169.00 (41.12%)	45.00 (32.85%)	52.00 (37.96%)	72.00 (52.55%)	0.003
Albumin, n (p%)	295.00 (71.78%)	111.00 (81.02%)	103.00 (75.18%)	81.00 (59.12%)	<0.001
Sedation Analgesia, n (p%)	356.00 (86.62%)	125.00 (91.24%)	119.00 (86.86%)	112.00 (81.75%)	0.070
Immunos, n (p%)	12.00 (2.92%)	5.00 (3.65%)	4.00 (2.92%)	3.00 (2.19%)	0.933
Infection Site					
Brain, n (p%)	20.00 (4.87%)	5.00 (3.65%)	3.00 (2.19%)	12.00 (8.76%)	0.030
Lung, n (p%)	325.00 (79.08%)	103.00 (75.18%)	110.00 (80.29%)	112.00 (81.75%)	0.373
Abdomen, n (p%)	98.00 (23.84%)	36.00 (26.28%)	37.00 (27.01%)	25.00 (18.25%)	0.168
Urinary, n (p%)	113.00 (27.49%)	29.00 (21.17%)	46.00 (33.58%)	38.00 (27.74%)	0.071
Other, n (p%)	46.00 (11.19%)	14.00 (10.22%)	21.00 (15.33%)	11.00 (8.03%)	0.145
Pathogen					
Escherichia_coli, n (p%)	45.00 (10.95%)	21.00 (15.33%)	8.00 (5.84%)	16.00 (11.68%)	0.040
Klebsiella_pneumoniae, n (p%)	51.00 (12.41%)	15.00 (10.95%)	20.00 (14.60%)	16.00 (11.68%)	0.625
Carbapenem-resistant K. pneumoniae, n (p%)	18.00 (4.38%)	3.00 (2.19%)	9.00 (6.57%)	6.00 (4.38%)	0.208
Acinetobacter_baumannii, n (p%)	94.00 (22.87%)	25.00 (18.25%)	35.00 (25.55%)	34.00 (24.82%)	0.285
Carbapenem-resistant A. baumannii, n (p%)	76.00 (18.49%)	19.00 (13.87%)	29.00 (21.17%)	28.00 (20.44%)	0.230
Pseudomonas_aeruginosa, n (p%)	54.00 (13.14%)	14.00 (10.22%)	19.00 (13.87%)	21.00 (15.33%)	0.435
Carbapenem-resistant P. aeruginosa, n (p%)	23.00 (5.60%)	7.00 (5.11%)	7.00 (5.11%)	9.00 (6.57%)	0.832
Staphylococcus_aureus, n (p%)	32.00 (7.79%)	8.00 (5.84%)	12.00 (8.76%)	12.00 (8.76%)	0.581
Methicillin-resistant S. aureus, n (p%)	7.00 (1.70%)	2.00 (1.46%)	3.00 (2.19%)	2.00 (1.46%)	0.999
Staphylococcus, n (p%)	87.00 (21.17%)	27.00 (19.71%)	27.00 (19.71%)	33.00 (24.09%)	0.592
Methicillin-resistant Staphylococcus n (p%)	33.00 (8.03%)	15.00 (10.95%)	7.00 (5.11%)	11.00 (8.03%)	0.206
Candida, n (p%)	175.00 (42.58%)	53.00 (38.69%)	64.00 (46.72%)	58.00 (42.34%)	0.404
Aspergillus fumigatus, n (p%)	22.00 (5.35%)	10.00 (7.30%)	6.00 (4.38%)	6.00 (4.38%)	0.464
Fungus, n (p%)	202.00 (49.15%)	59.00 (43.07%)	77.00 (56.20%)	66.00 (48.18%)	0.090
Gram positive Cocci, n (p%)	241.00 (58.64%)	81.00 (59.12%)	83.00 (60.58%)	77.00 (56.20%)	0.755
Gram positive Bacilli, n (p%)	53.00 (12.90%)	19.00 (13.87%)	12.00 (8.76%)	22.00 (16.06%)	0.181
Gram negative Bacilli, n (p%)	291.00 (70.80%)	95.00 (69.34%)	99.00 (72.26%)	97.00 (70.80%)	0.868
Clinical Outcomes					
Los icu, day	9.00 (4.50 - 17.60)	6.50 (2.70 - 13.00)	10.10 (6.00 - 19.90)	10.00 (5.60 - 18.70)	<0.001
ICU mortality, n (%)	84.00 (20.44%)	36.00 (26.28%)	25.00 (18.25%)	23.00 (16.79%)	0.111
14-Day All-Cause Mortality, n (%)	143.00 (34.79%)	68.00 (49.64%)	48.00 (35.04%)	27.00 (19.71%)	<0.001
28-Day All-Cause Mortality, n (%)	194.00 (47.20%)	87.00 (63.50%)	59.00 (43.07%)	48.00 (35.04%)	<0.001

### 3.2 Kaplan-Meier survival curves

**Figure 2:**
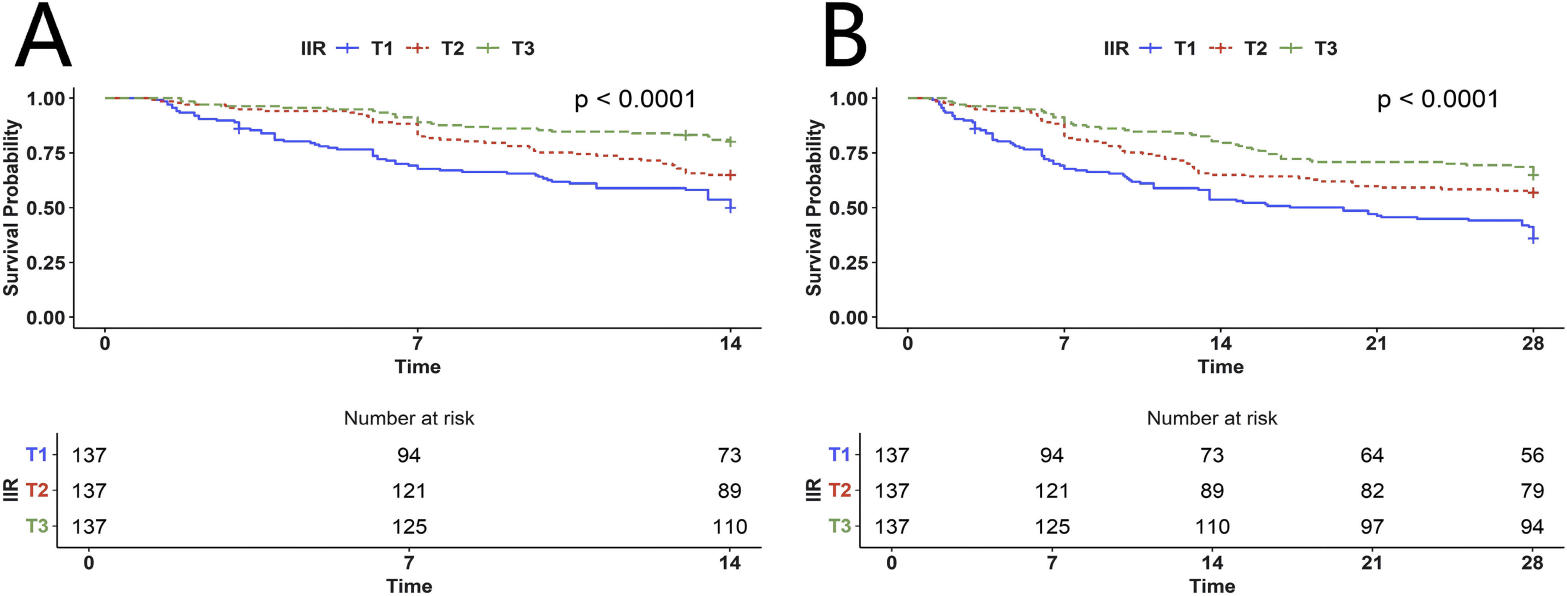
Kaplan-Meier curves and cumulative incidence of 14-day **(A)** and 28-day **(B)** all-cause mortality stratified by IIR groups. Patients in the higher IIR tertiles had significantly better survival than those in the lowest tertile, with all log-rank P values < 0.001. The number-at-risk table shows the number of patients remaining under observation at each time point.

Overall, 143 patients died within 14 days and 194 died within 28 days. Kaplan-Meier survival curves showed that, compared with patients in the IIR T1 group, those in the T2 and T3 groups had significantly lower 14-day and 28-day all-cause mortality (**Figure 2**, all log-rank P < 0.001).

### 3.3 Association between IIR levels and clinical outcomes

**Figure 3:**
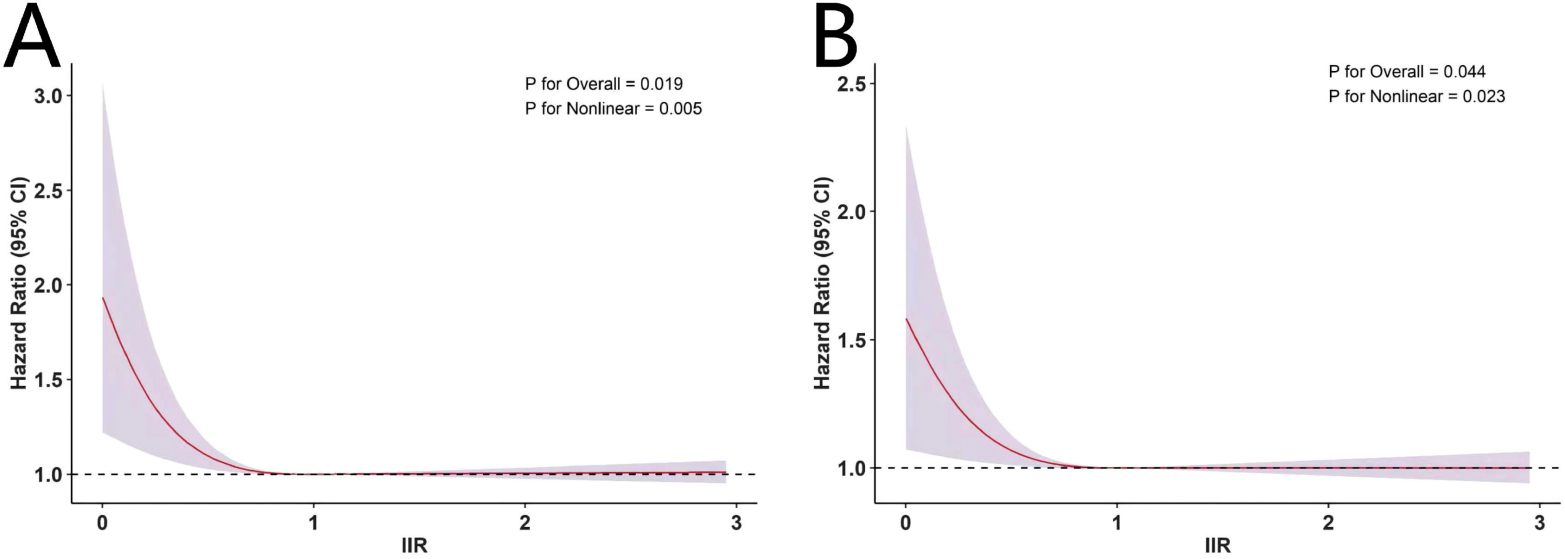
Restricted cubic spline regression analysis of IIR with 14-day **(A)** and 28-day **(B)** all-cause mortality in patients with sepsis. IIR showed a nonlinear association with both outcomes, with P for nonlinearity < 0.05.

Boruta feature selection and LASSO regression identified eight common potential risk factors: mean arterial pressure, SOFA score, APACHE II score, total bilirubin, anion gap, vasopressor use, antihypertensive therapy, and Candida infection (**Figures 2**, **3**). These potential risk factors were included in multivariable Cox regression models to evaluate the association between IIR and all-cause mortality in patients with sepsis. In the unadjusted model (Model 1), higher IIR levels were closely associated with reduced all-cause mortality risk, including 14-day mortality (HR = 0.31, 95% CI: 0.20–0.49, P < 0.001) and 28-day mortality (HR = 0.41, 95% CI: 0.29–0.59, P < 0.001). In Model 2, after adjustment for age, gender, mean arterial pressure, SOFA score, APACHE II score, total bilirubin, anion gap, vasopressor use, antihypertensive therapy, and Candida infection, higher IIR levels remained associated with a lower risk of all-cause mortality: 14-day mortality (HR = 0.48, 95% CI: 0.30–0.77, P < 0.001) and 28-day mortality (HR = 0.62, 95% CI: 0.42–0.90, P < 0.001) (**Table 2**).

**Table 2:** Cox regression model.

	Model 1		Model 2	
	HR (95% CI)	P value	HR (95% CI)	P value
14-day all-cause mortality				
T1	Reference		Reference	
T2	0.60(0.42-0.87)	0.007	0.76(0.56-0.95)	0.037
T3	0.31(0.20-0.49)	<0.001	0.48(0.30-0.77)	0.002
P for trend	<0.001		<0.001	
28-day all-cause mortality				
T1	Reference		Reference	
T2	0.56(0.40-0.78)	0.001	0.73(0.51-1.02)	0.066
T3	0.41(0.29-0.59)	<0.001	0.62(0.42-0.90)	0.012
P for trend	<0.001		0.007	

Model 1: Unadjusted;

Model 2: Adjusted age, gender, mean arterial pressure, SOFA score, APACHE II score, total bilirubin, anion gap, vasopressor use, antihypertensive therapy, and Candida infection.

### 3.4 Nonlinear relationship between IIR and mortality

Restricted cubic spline analysis demonstrated a nonlinear association between IIR and both 14-day and 28-day all-cause mortality (all P for nonlinearity < 0.05, **Figure 3**). The two-piecewise Cox proportional hazards model identified inflection points of 0.066 for 14-day mortality and 0.073 for 28-day mortality. Below these thresholds, each 0.01-unit increase in IIR was associated with a 21% reduction in 14-day mortality risk (HR = 0.79, 95% CI: 0.68–0.91) and a 40% reduction in 28-day mortality risk (HR = 0.60, 95% CI: 0.43–0.98). Likelihood ratio tests confirmed that these associations were statistically significant (P < 0.05, **Table 3**).

**Table 3:** Two-piecewise Cox proportional model.

	Adjusted HR (95% CI)	p value
14-day all-cause mortality		
Inflection point	0.066	
IIR<0.066	0.79(0.68-0.91)	0.008
IIR≥0.066	1.00(0.99-1.00)	0.892
p for likelihood ratio test		0.006
28-day all-cause mortality		
Inflection point	0.073	
IIR<0.073	0.60(0.43-0.98)	0.004
IIR≥0.073	0.99(0.99-1.00)	0.661
p for likelihood ratio test		0.008

### 3.5 ROC curve analysis

**Figure 4:**
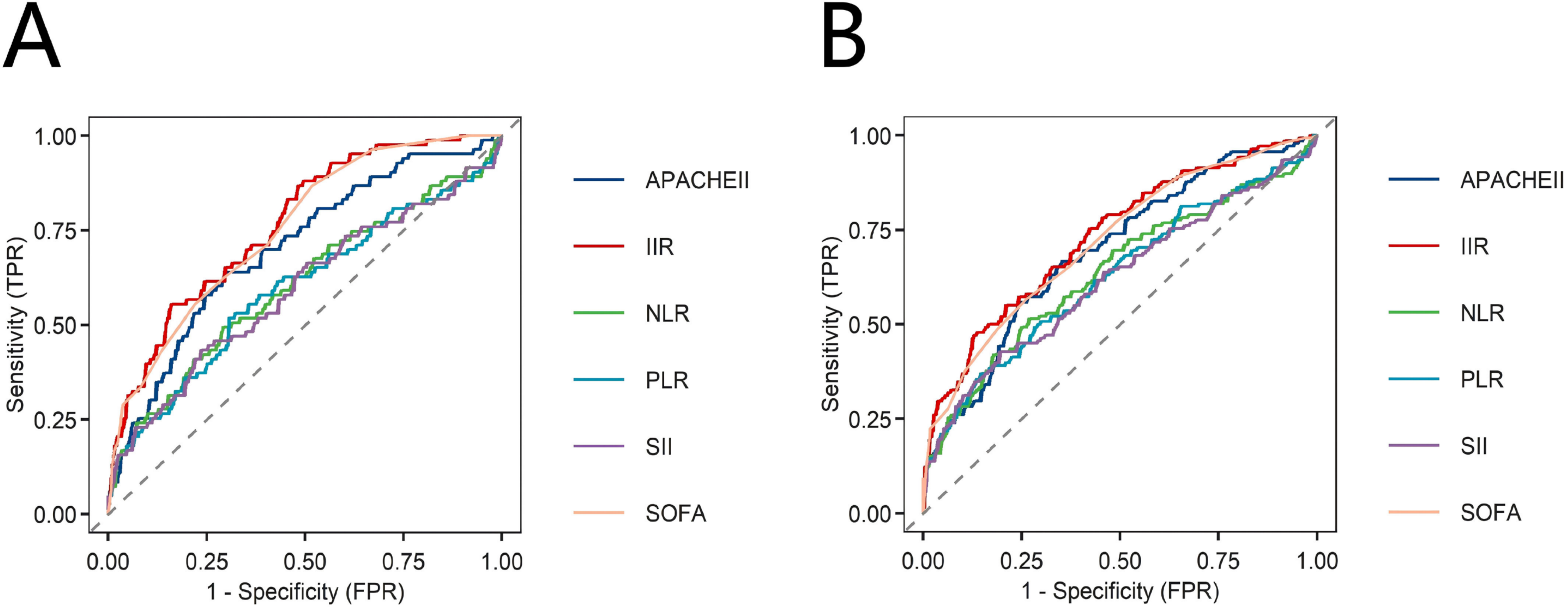
Receiver operating characteristic curves assesses the predictive capability of the IIR index for 14-day **(A)** and 28-day **(B)** all-cause mortality. IIR showed discriminatory ability, with AUCs of 0.77 for 14-day mortality and 0.73 for 28-day mortality. These values were not inferior to that of SOFA and APACHE II scores and superior to those of neutrophil-to-lymphocyte ratio, systemic immune-inflammation index, and platelet-to-lymphocyte ratio (detailed results are shown in **Table 4**).

**Figure 5:**
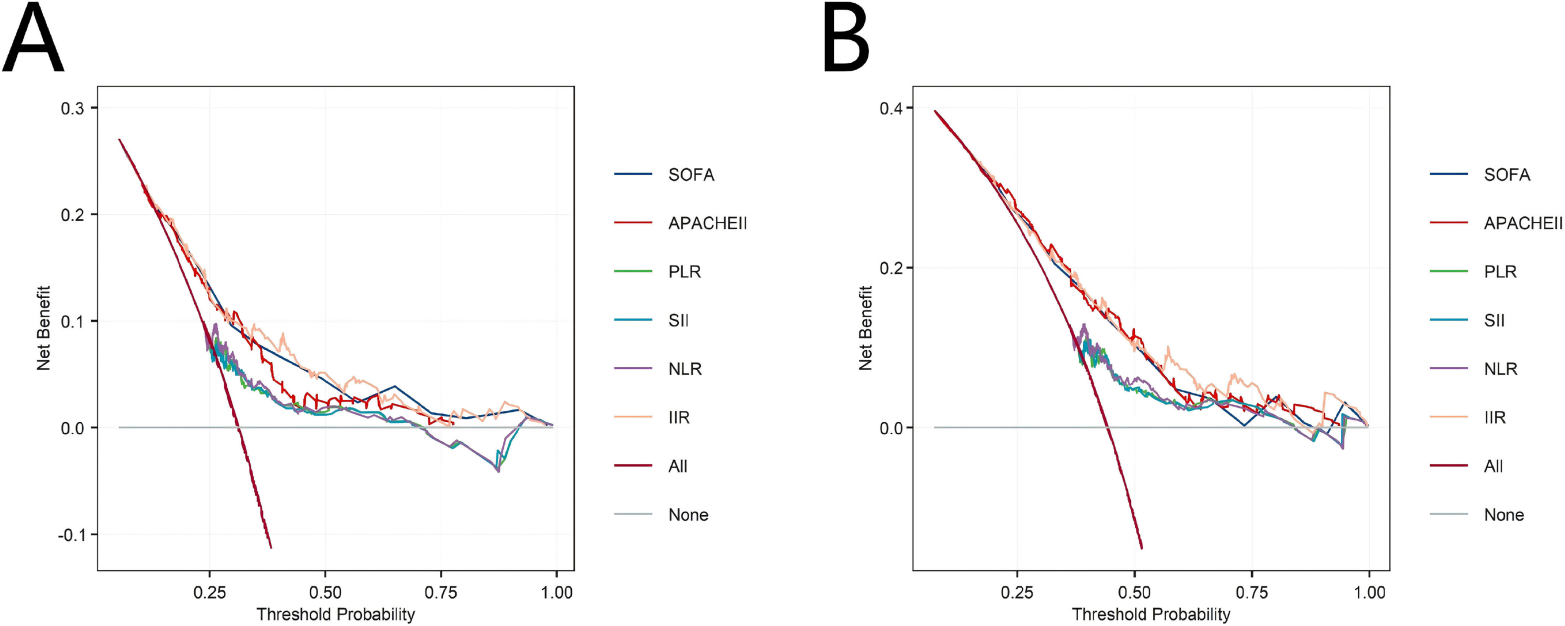
DCA curves curves of the IIR index for 14-day **(A)** and 28-day **(B)** all-cause mortality. IIR showed a net benefit comparable to SOFA score and higher than APACHE II, NLR, SII, and PLR across selected threshold probabilities. The “All” and “None” lines represent the strategies of treating all or no patients as high risk, respectively.

**Figure 6:**
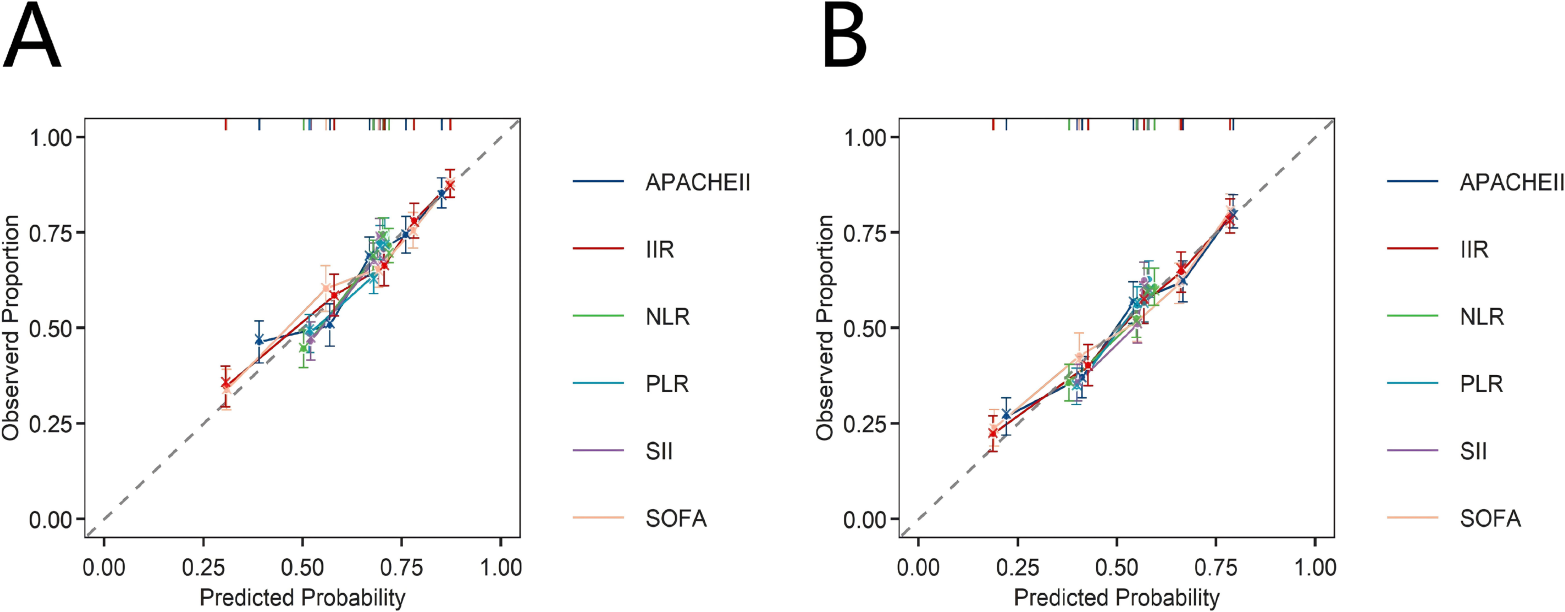
Calibration curves curves of the IIR index for 14-day **(A)** and 28-day **(B)** all-cause mortality. The fitted curves for IIR and SOFA score were closest to the reference diagonal line, indicating better agreement between predicted and observed mortality risks. These results reflect internal calibration and require external validation.

ROC curves were generated for IIR, SOFA score, APACHE II score, neutrophil-to-lymphocyte ratio, systemic immune-inflammation index, and platelet-to-lymphocyte ratio to compare their predictive value for 14-day and 28-day all-cause mortality. For 14-day mortality, the AUC of IIR was 0.77 (95% CI: 0.71–0.82). For 28-day mortality, the AUC was 0.73 (95% CI: 0.68–0.78). These values were not inferior to that of SOFA and APACHE II scores and superior to those of neutrophil-to-lymphocyte ratio, systemic immune-inflammation index, and platelet-to-lymphocyte ratio (**Figure 4**, detailed results are shown in **Table 4**). Decision curve analysis showed that the net benefit of IIR-guided prediction was comparable to that of the SOFA score and higher than those of APACHE II score, neutrophil-to-lymphocyte ratio, systemic immune-inflammation index, and platelet-to-lymphocyte ratio (**Figure 5**). Calibration curves indicated that the fitted curves for IIR and SOFA score were closest to the reference diagonal line, outperforming APACHE II score, neutrophil-to-lymphocyte ratio, systemic immune-inflammation index, and platelet-to-lymphocyte ratio (**Figure 6**).

**Table 4:** Information of ROC curves in **Figure 4**.

	AUC	95% CI	Sensitivity	Septicity
14-day all-cause mortality				
IIR	0.77	0.71-0.82	0.84	0.55
SOFA	0.75	0.70-0.81	0.87	0.48
APACHE II	0.70	0.64-0.76	0.70	0.64
NLR	0.60	0.53-0.67	0.71	0.49
SII	0.59	0.52-0.66	0.76	0.43
PLR	0.60	0.52-0.67	0.69	0.52
28-day all-cause mortality				
IIR	0.73	0.68-0.78	0.87	0.47
SOFA	0.72	0.67-0.77	0.74	0.56
APACHE II	0.69	0.63-0.75	0.76	0.55
NLR	0.64	0.58-0.69	0.73	0.51
SII	0.62	0.55-0.68	0.81	0.42
PLR	0.63	0.56-0.69	0.85	0.37

### 3.6 Subgroup analysis

**Figure 7:**
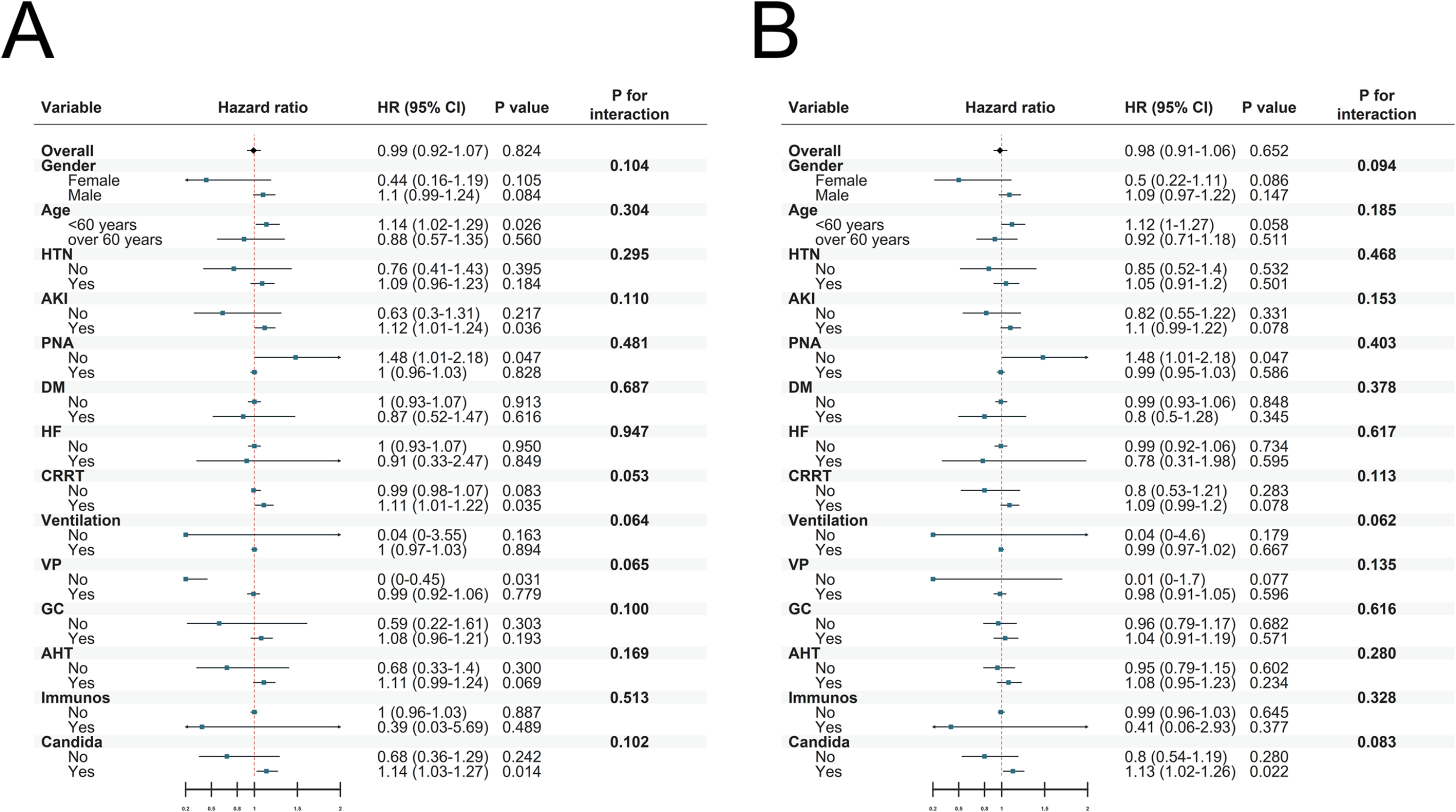
Forest plots of stratified analyses of IIR (continuous) and 14-day **(A)** and 28-day **(B)** all-cause mortality in patients. No significant interaction was observed across subgroups, suggesting a generally consistent association between IIR and mortality risk.

The association between IIR and the risk of 14-day and 28-day all-cause mortality was further examined across different patient subgroups. Stratified analyses according to sex, age, hypertension, acute kidney injury, pneumonia, diabetes, heart failure, continuous renal replacement therapy, mechanical ventilation, vasoactive agents, corticosteroids, antihypertensive drugs, immunosuppressants, and Candida infection showed no statistically significant effect modification (all P for interaction > 0.05, **Figure 7**).

## 4 Discussion

Immune dysregulation is a central contributor to the high mortality associated with sepsis (3). During the early phase of sepsis, pathogen-associated molecular patterns and damage-associated molecular patterns activate the innate immune system and trigger a cascade of inflammatory responses. This process results in excessive release of pro-inflammatory cytokines, including TNF-α, interleukin-1β, and IL-6, forming what is commonly described as a cytokine storm (18). Although this inflammatory response is intended to eliminate invading pathogens, uncontrolled inflammation can lead to tissue injury, microcirculatory dysfunction, and multiple organ dysfunction. As sepsis progresses, the host may enter a compensatory anti-inflammatory response phase, characterized by immune paralysis or immunosuppression (13). This stage is marked by excessive anti-inflammatory cytokine expression, impaired immune cell function, and apoptosis (19). Sepsis-related immunopathology also involves disruption of T helper cell subset balance. Under physiological conditions, Th1 cells, which mediate cellular immunity, and Th2 cells, which mediate humoral immunity, remain in dynamic equilibrium (20). In sepsis, this balance is disturbed and often shifts toward a Th2-dominant response, a pattern closely related to patient prognosis (21, 22).

IFN-γ and IL-10 are central cytokines in sepsis immunoregulation, and their balance may influence the overall direction of the immune response.

IFN-γ is a key pro-inflammatory cytokine produced mainly by activated T cells and natural killer cells. It links innate and adaptive immunity, enhances macrophage bactericidal activity, induces MHC class I molecule expression, and plays an essential role in clearing viral and bacterial infections (12, 23). Previous studies have shown that IFN-γ can promote the Warburg effect through the PI3K-AKT-mTOR pathway, thereby reversing immunosuppression in septic mice (12). In patients with sepsis, blood IFN-γ levels greater than 3 pg/mL have been used to define an “IFN-γ-driven sepsis” endotype, which is independently associated with increased mortality risk. Conversely, markedly reduced IFN-γ levels are associated with an immunosuppressive phenotype characterized by decreased HLA-DR expression and increased PD-L1 and IL-10 levels (23).

IL-10 is a potent anti-inflammatory cytokine that suppresses pro-inflammatory cytokine production and helps prevent tissue damage caused by excessive inflammation (8, 24). However, sustained overproduction of IL-10 is one of the major risk factors for sepsis severity and poor prognosis (25). Studies have shown that plasma IL-10 levels in patients with sepsis parallel disease severity, and high IL-10 levels are associated with worse survival outcomes (26, 27).

Imbalance between IFN-γ and IL-10 may contribute to immune dysfunction (8). Giovanni et al. (28) demonstrated that patients with sepsis had significantly decreased IFN-γ levels and persistently elevated IL-10 levels, a pattern associated with poor prognosis. A higher IFN-γ/IL-10 ratio may therefore reflect a more activated immune state and stronger anti-infective potential, whereas a lower ratio may indicate immune suppression and impaired anti-infective capacity. This balance appears closely related to clinical outcomes. *In vitro* experiments have shown that when bacterial lysates are added to healthy human blood to simulate infection, stronger pathogen stimulation is accompanied by a lower IFN-γ/IL-10 ratio (17). Findings from septic mouse models and clinical data support this observation, showing that patients with sepsis have significantly lower IFN-γ/IL-10 ratios than patients with non-septic systemic inflammatory response syndrome (17, 29). These findings suggest that a low ratio may reflect a greater infectious burden.

In the present study, increased IIR levels were closely associated with reduced all-cause mortality risk in patients with sepsis. Further analyses showed a clear nonlinear relationship between IIR and mortality risk, with inflection points of 0.066 and 0.073 identified by the two-piecewise Cox model. Below these thresholds, each 0.01-unit increase in IIR was associated with 21% and 60% reductions in 14-day and 28-day mortality risk, respectively. Once IIR exceeded these thresholds, the protective association with mortality tended to plateau. This pattern may reflect an immune equilibrium point during the host response to infection. Values below the threshold suggest relative dominance of IL-10-mediated anti-inflammatory responses, leading to suppression of Th1-type cellular immunity, reduced macrophage activation, impaired antigen presentation, and inadequate pathogen clearance. Sustained high IL-10 expression may also induce lymphocyte apoptosis, pushing the host toward an immune paralysis state and increasing the risk of secondary infection and death. Such patients may require closer dynamic immune monitoring and, when appropriate, consideration of immunostimulatory therapy or cautious reassessment of immunosuppressive agents such as corticosteroids to avoid aggravating immunosuppression. Above the threshold, further increases in IFN-γ may no longer provide additional benefit and may even contribute to tissue injury through excessive inflammation. This dynamic balance suggests that the IFN-γ/IL-10 ratio may have optimal predictive value within a specific range. The observed nonlinear relationship also points to potential biological saturation or compensatory feedback mechanisms, emphasizing the importance of considering complex dose-response patterns in precision immunomodulation.

This study found that IIR showed favorable performance in predicting prognosis among patients with sepsis. For 14-day and 28-day mortality, the AUC values reached 0.75 and 0.71, respectively. Its predictive ability was not inferior to that of SOFA and APACHE II scores and was superior to traditional inflammatory indices, including neutrophil-to-lymphocyte ratio, systemic immune-inflammation index, and platelet-to-lymphocyte ratio. SOFA and APACHE II scores have long been used to evaluate disease severity and organ dysfunction in critically ill patients (30, 31), while neutrophil-to-lymphocyte ratio, systemic immune-inflammation index, and platelet-to-lymphocyte ratio are commonly used clinical indices for assessing inflammation and immune status (32–35). Although PLR showed between-group differences in baseline characteristics and was weakly correlated with IIR, its independent prognostic value after multivariable adjustment was substantially weaker than that of IIR, which does not support replacing IIR with PLR for prognostic stratification(**Supplementary Tables 4**, **5**). These tools remain important for prognostic evaluation. Our findings suggest that IIR may have practical value for more rapid prognostic assessment in patients with sepsis. The association was also stable across all analyzed subgroups.

Given its prognostic value, dynamic changes in the IFN-γ/IL-10 ratio may provide useful information for immunomodulatory strategies in sepsis. Patients with sepsis and immunosuppression often show a decreased IFN-γ/IL-10 ratio, and IFN-γ supplementation may represent a potential therapeutic approach (36, 37). IFN-γ release assays have been used to monitor T lymphocyte function in patients with septic shock and have shown a significant association between reduced IFN-γ release capacity and absolute CD8 lymphocyte counts (37). However, clinical trials of IFN-γ supplementation have yielded inconsistent results, possibly due to insufficient patient selection and lack of immune status stratification (17, 36). The IFN-γ/IL-10 ratio may serve as a biomarker for immune status assessment, helping to identify immunosuppressed patients who may benefit from IFN-γ-enhancing therapy or guiding the use of immunomodulatory agents such as corticosteroids (17). Conversely, for patients in a hyperinflammatory state, targeted inhibition of IFN-γ or enhancement of IL-10 may be more appropriate.

Future studies should focus on dynamic monitoring of the IFN-γ/IL-10 ratio and explore its clinical significance at different stages of sepsis. For example, a low baseline IFN-γ response may predict hospitalization risk in patients with COVID-19 (38). Larger prospective studies are needed to validate the effectiveness of the IFN-γ/IL-10 ratio in guiding individualized immunotherapy, especially in patient subgroups for whom current treatment strategies remain insufficient. Potential approaches include targeting immune checkpoints, such as PD-1/PD-L1, or modulating cellular metabolic pathways, such as the Warburg effect, to reverse immune dysregulation (12, 39, 40).

This study has several limitations. As a single-center retrospective study, the generalizability of the findings may be limited. Large-scale, multicenter, prospective randomized controlled trials are needed to determine whether treatment strategies based on this ratio can improve clinical endpoints. In addition, the present analysis focused on the initial IIR level at admission and did not evaluate its dynamic changes over time. Future studies should systematically assess the predictive value of IIR at different time points, such as admission, 48 h, and 7 days, as well as the prognostic significance of its rate of change. The exact duration of illness before enrollment was not clearly defined, preventing precise differentiation of immune phases in patients with sepsis. Therefore, although this study confirmed a nonlinear association between IIR and mortality risk, causality cannot be established. Despite its promising prognostic potential, the lack of a unified threshold limits the clinical application of IIR. The optimal detection window should be clarified, and standardized cutoff values should be established through multicenter studies to enable reliable application across centers.

Despite these limitations, this study has important clinical implications. As a composite immune marker, IIR provides a quantitative measure for assessing immune status in patients with sepsis. Its underlying pathophysiological mechanisms and clinical applicability warrant further investigation. This ratio may serve as a candidate biomarker for immune stratification and trial enrollment, although further validation and standardization are needed across different centers, time windows, and interventional settings. Future research may further explore the effects of IIR on immune cell metabolic reprogramming and epigenetic regulation at different stages of sepsis, with the aim of supporting more precise immune phenotyping and individualized risk assessment.

## 5 Conclusion

The IFN-γ/IL-10 ratio was independently associated with short-term mortality in patients with sepsis and showed modest discriminatory ability. These findings suggest that IIR may be a candidate biomarker for immune-inflammatory risk stratification. However, external validation and prospective studies are required before clinical implementation.

## Data Availability

The raw data supporting the conclusions of this manuscript will be made available by the authors, without undue reservation, to any qualified researcher, further inquiries can be directed to the corresponding author/s.
