# Supplementary material for: Interferon-gamma to interleukin-10 ratio is negatively associated with short-term mortality risk in sepsis patients: a retrospective study based on immune balance index
Source: Front Immunol. 2026 Jul 28;17:1816500. doi: 10.3389/fimmu.2026.1816500 (PMC13457149; doi:10.3389/fimmu.2026.1816500)
Supplement: Supplementary file 1 [file DataSheet1.docx]

# 1 Source of Laboratory Data and Detection Methods for Key Factors

All biochemical data in this study were obtained from routine clinical laboratory records in the hospital electronic medical record system and laboratory information management system. The measurements were performed using venous blood samples collected within 24 h after ICU admission as part of standard clinical care.

Assay platform and specimen type

Cytokine concentrations were measured using a LEGENDplex bead-based multiplex immunoassay, with data acquisition performed on a BD FACSCanto II flow cytometer. Serum specimens collected in serum separator tubes were analyzed with the LEGENDplex multiplex cytokine assay kit.IIR values for all samples were calculated directly based on the measured numeric values, without the need for imputation or substitution.The assay platform used in our study has higher sensitivity than conventional ELISA, with detection sensitivities of 0.007 pg/mL for IFN-γ and 0.02 pg/mL for IL-10. Therefore, reliable quantitative values could still be obtained near the theoretical lower limit of 0 pg/mL. The present study used the LegendPlex multiplex assay to simultaneously measure 12 cytokines, including IFN-γ, IL-10, IL-4, IL-6, IL-8, IL-12, and IL-17. IFN-γ and IL-10 were the core indicators of interest in this study, while the remaining cytokines were collected as exploratory data.

Sampling time

Measurement of IFN-γ and IL-10 within 24 hours after ICU admission was required for study inclusion. For patients with repeated measurements during this period, the first available value was used for analysis.

Measurement range and lower limit of detection

In patients with sepsis, serum IFN-γ levels generally ranged from 0 to 50 pg/mL, while IL-10 levels generally ranged from 0 to 500 pg/mL.The assay platform used in our study has higher sensitivity than conventional ELISA, with detection sensitivities of 0.007 pg/mL for IFN-γ and 0.02 pg/mL for IL-10.

Handling of values below the detection threshold

Values below the lower limit of detection were imputed as LLOD/√2 in the primary statistical analysis. In sensitivity analyses, these values were alternatively treated as the LOD value and as zero to evaluate the influence of this handling strategy on the IFN-γ/IL-10 ratio and the study conclusions.

Laboratory quality control procedures

The data were derived from routine clinical testing performed by the hospital laboratory. All relevant quality control procedures strictly followed the standardized operating procedures of the hospital’s clinical laboratory department and the requirements of the National Health Commission of China.

# 2 Supplementary Figures and Tables（Description of Statistical Methods）

The extent of missing data for each variable.(sTable 1)

sTable 1

Proportion of missing data for variables

| **Variable** | **Proportion of missing data,%** |  | **Variable** | **Proportion of missing data,%** |
| --- | --- | --- | --- | --- |
| age | 0 |  | Anion gap | 1.0 |
| **Scores** | 0 |  | Calcium | 0.7 |
| SOFA Score | 0 |  | Potassium | 0.7 |
| APACHE II Score | 0 |  | Sodium | 0.7 |
| **Vital Signs** | 0 |  | Glucose | 0.7 |
| Heart Rate | 0 |  | Neutrophil gelatinase associated lipocalin | 6.6 |
| Respiratory Rate | 0.2 |  | Heparin binding protein | 5.1 |
| Systolic Blood Pressure | 0 |  | Lactate | 0 |
| Diastolic Blood Pressure | 0 |  | Creatine Kinase | 4.9 |
| Mean Arterial Pressure | 0 |  | Creatine Kinase MB | 4.9 |
| Temperature | 0.2 |  | Hs-cTnT | 6.1 |
| **Laboratory parameters** | 0 |  | proBNP | 12.2 |
| White blood cells | 0 |  | PCT | 1.2 |
| Red blood cells | 0 |  | IL6 | 3.6 |
| Hemoglobin | 0 |  | IL4 | 0 |
| Platelets | 0 |  | IL8 | 0 |
| Neutrophil cells | 0 |  | IL10 | 0 |
| Lymphocyte cells | 0 |  | IL17 | 0 |
| Total bilirubin | 1.0 |  | IFN-α | 0 |
| Direct bilirubin | 1.7 |  | IFN-γ | 0 |
| Indirect bilirubin | 1.9 |  | International normalized ratio | 0.7 |
| Albumin | 1.0 |  | Prothrombin time | 0.7 |
| Alanine aminotransferase | 0 |  | Partial thromboplastin | 0.7 |
| Aspartate aminotransferase | 0 |  | pH | 0 |
| Creatinine | 0.7 |  | PaO_2_ | 0 |
| Blood urea | 1.5 |  | PCO_2_ | 0 |

Imputation method: Multiple imputation was used to handle missing data. Given that the highest missingness was 12.2%, we performed 20 imputations to ensure adequate statistical efficiency.

Winsorization strategy: Although winsorization was prespecified, no variables ultimately required winsorization after data checking; therefore, the final analyses were conducted using the original values.

Variable selection for multivariable models: Variable selection was performed in two stages. In the first stage, Boruta feature selection was applied with a maximum of 300 iterations. The Boruta screening results for all variables has been presented in Supplementary sFigure 1. In the second stage, LASSO regression was used for dimensionality reduction. Ten-fold cross-validation was applied, and the more conservative λ_1se was selected. The LASSO coefficient path plot, showing changes in variable coefficients across different values of λ, has been presented in sFigure 2. IIR was the primary exposure variable and was therefore retained in all Cox models a priori, regardless of the results of automated variable selection. Boruta and LASSO were used to select adjustment covariates from candidate clinical and laboratory variables. In addition to data-driven selection, clinically important variables known to be associated with sepsis prognosis were reviewed by the study team. Variables considered essential for confounding control were retained even if their statistical contribution was modest. The final covariates selected for adjustment were mean arterial pressure, SOFA score, APACHE II score, total bilirubin, anion gap, vasopressor use, antihypertensive therapy, and Candida infection.

sFigure 1 Boruta feature selection. The Boruta algorithm was used to identify relevant candidate predictors among clinical, laboratory, and treatment variables. Variables classified as “Confirmed” were considered important predictors, while shadow variables served as randomized reference benchmarks. IIR was the primary exposure variable and was retained a priori in the multivariable models.

sFigure 2 LASSO regression. Cross-validation curve showing the selection of the optimal penalty parameter lambda; the numbers at the top indicate the number of variables with non-zero coefficients.(left) Coefficient profile plot showing changes in variable coefficients across different lambda values. (right)

Assessment of the Cox proportional hazards assumption: The proportional hazards assumption was evaluated using Schoenfeld residuals, including both global and variable-specific tests.

Parameter settings for restricted cubic spline analysis: Restricted cubic spline analysis was performed using four knots, as recommended by Harrell. The goodness of fit between the linear model and the spline model was compared using the likelihood ratio test to determine whether a significant nonlinear exposure-response relationship was present.

Time-dependent ROC curves were used to evaluate discrimination for 14-day and 28-day mortality.

Calibration was assessed by comparing predicted and observed risks using bootstrap resampling with 1,000 repetitions.

Decision curve analysis was performed to evaluate the net benefit across clinically relevant threshold probabilities.

Parameter settings for threshold effect analysis: Threshold effects were assessed using a two-piecewise linear regression model. The search range was restricted to the 10th to 90th percentiles of the exposure variable to avoid false-positive findings driven by extreme values. The 95% confidence interval for the estimated threshold was obtained using the bootstrap method, and the likelihood ratio test was applied.

sTable 2 Cox regression model

Model 1: Adjusted Acute Physiology and Chronic Health II;

Model 2: Adjusted sequential organ failure assessment.

|  | Model 1 | |  | Model 2 | |
| --- | --- | --- | --- | --- | --- |
|  | HR (95% CI) | P value |  | HR (95% CI) | P value |
| 14-day all-cause mortality |  |  |  |  |  |
| T1 | Reference |  |  | Reference |  |
| T2 | 0.81(0.65-0.99) | 0.048 |  | 0.88(0.68-1.10) | 0.068 |
| T3 | 0.44(0.28-0.71) | 0.001 |  | 0.47(0.29-0.75) | 0.002 |
| P for trend | <0.001 | |  | 0.003 | |
|  |  | |  |  | |
| 28-day all-cause mortality |  |  |  |  |  |
| T1 | Reference |  |  | Reference |  |
| T2 | 0.71(0.50-0.99) | 0.045 |  | 0.72(0.50-1.00) | 0.05 |
| T3 | 0.51(0.35-0.74) | <0.001 |  | 0.61(0.42-0.89) | 0.01 |
| P for trend | 0.007 | |  | 0.01 | |

sTable 3 multivariable Cox regression analysis without imputation of missing values

|  | Model 1 | |  | Model 2 | |
| --- | --- | --- | --- | --- | --- |
|  | HR (95% CI) | P value |  | HR (95% CI) | P value |
| 14-day all-cause mortality |  |  |  |  |  |
| T1 | Reference |  |  | Reference |  |
| T2 | 0.60(0.42-0.87) | 0.007 |  | 0.76(0.56-0.95) | 0.037 |
| T3 | 0.31(0.20-0.49) | <0.001 |  | 0.48(0.30-0.77) | 0.002 |
| P for trend | <0.001 | |  | <0.001 | |
|  |  | |  |  | |
| 28-day all-cause mortality |  |  |  |  |  |
| T1 | Reference |  |  | Reference |  |
| T2 | 0.56(0.40-0.78) | 0.001 |  | 0.73(0.51-1.02) | 0.066 |
| T3 | 0.41(0.29-0.59) | <0.001 |  | 0.62(0.42-0.90) | 0.012 |
| P for trend | <0.001 | |  | 0.007 | |

Model 1: Unadjusted;

Model 2: Adjusted age, gender, mean arterial pressure, SOFA score, APACHE II score, total bilirubin, anion gap, vasopressor use, antihypertensive therapy, and Candida infection

sTable 4 Correlation analysis between PLR and IIR

| **Variable** | **Variable** | **P value** | **rho** |
| --- | --- | --- | --- |
| PLT | IIR | 0.001 | 0.1886 |

sTable 5 multivariable Cox regression analysis with PLR

Model 1: Unadjusted;

Model 2: Adjusted age, gender, mean arterial pressure, SOFA score, APACHE II score, total bilirubin, anion gap, vasopressor use, antihypertensive therapy, and Candida infection

|  | Model 1 | |  | Model 2 | |
| --- | --- | --- | --- | --- | --- |
|  | HR (95% CI) | P value |  | HR (95% CI) | P value |
| 14-day all-cause mortality |  |  |  |  |  |
| T1 | Reference |  |  | Reference |  |
| T2 | 1.08(0.71-1.65) | 0.724 |  | 1.14(0.73-1.77) | 0.564 |
| T3 | 1.24(0.82-1.86) | 0.305 |  | 1.31(0.85-2.01) | 0.222 |
| P for trend | 0.067 | |  | 0.118 | |
|  |  | |  |  | |
| 28-day all-cause mortality |  |  |  |  |  |
| T1 | Reference |  |  | Reference |  |
| T2 | 1.01(0.67-1.37) | 0.805 |  | 1.07(0.74-1.53) | 0.681 |
| T3 | 1.14(0.75-1.54) | 0.633 |  | 1.20(0.86-1.71) | 0.367 |
| P for trend | 0.169 | |  | 0.312 | |
